# 

**Published:** 1893-10

**Authors:** 


### CONTENTS.

PROCEEDINGS.

World's Columbian Dental Congress—1893 .............................. 469

Progress in Medical Education .............................................. 516

EDITORIAL.

The Pan-American Medical Congress.... ............... ................... 517

Bibliographical—The American Medical Temperance Quarterly...... 519

Bibliographical ........................................ ................ ............. 520

Obituary ........................................................................... 520

